# QTL analysis of seed germination traits in tobacco (*Nicotiana tabacum* L.)

**DOI:** 10.1007/s13353-021-00623-6

**Published:** 2021-03-05

**Authors:** Monika Agacka-Mołdoch, Mian Abdur Rehman Arif, Ulrike Lohwasser, Teresa Doroszewska, Ramsey S. Lewis, Andreas Börner

**Affiliations:** 1 Leibniz Institute of Plant Genetics and Crop Plant Research (IPK), Gatersleben, Germany; 2 Institute of Soil Science and Plant Cultivation, State Research Institute, Puławy, Poland; 3 Nuclear Institute for Agriculture and Biology (NIAB), Faisalabad, Pakistan; 4 Department of Crop and Soil Science, North Carolina State University, Raleigh, NC USA

**Keywords:** Seeds, Germination, Controlled deterioration, Storability, Genetic mapping

## Abstract

Genetic mapping of seed germination traits has been performed with many plant species. In tobacco, however, investigations are rare. In the present study, a bi-parental mapping population consisting of 118 doubled haploid lines and derived from a cross between ‘Beinhart-1000’ and ‘Hicks’ was investigated. Four germination-related traits, total germination (TG), normal germination (NG), time to reach 50% of total germination (T50), and the area under the curve after 200 h of germination (AUC) were considered by examining seeds either untreated or after a moderate controlled deterioration (CD). Quantitative trait loci were found for all traits distributed on 11 out of the 24 linkage groups. It was demonstrated that, as in many other species, germination-related traits are very complex and under polygenic control.

**Supplementary Information:**

The online version contains supplementary material available at 10.1007/s13353-021-00623-6.

## Introduction

A successful germination of seeds is the starting point of the growth cycle in higher plants. It is not only the germination *per se* that is important, however. The speed of germination also plays an important role because, during the germination process, the plantlets are very sensitive to biotic and abiotic stresses. Quick germination can therefore be advantageous. Both germination and germination speed typically decline with an increase in seed storage time.

This behavior has been demonstrated for tobacco (Agacka et al. 2013, 2014) in investigations of genebank accessions of *N. tabacum* L. and *N. rustica* L. stored at different temperatures. Reducing the temperature from 20 to 0 °C increased storability from about 10 to 30 years, and a further reduction to − 15/− 18 °C increased storability to more than 50 years. For materials stored at 20 °C, a significant effect of seed moisture content has been demonstrated.

However, the survival of seeds is determined not only by environmental conditions, but also by genetic factors. Molecular mapping of responsible loci has been carried out in several plant species including *Arabidopsis* (Bentsink et al. 2000; Clerkx et al. 2004), rice (Miura et al. 2002; Xue et al. 2008), soybean (Singh et al. 2008), barley (Nagel et al. 2009), lettuce (Schwember and Bradford 2010), oilseed rape (Nagel et al. 2011), and wheat (Landjeva et al. 2010; Rehman Arif and Börner 2020).

An initial mapping study of seed germination traits in tobacco was performed by Agacka-Mołdoch et al. (2015) who studied 122 recombinant inbred lines derived from a cross between the cultivars ‘Florida 301’ and ‘Hicks’. Four genomic regions located on four different linkage groups were identified to be associated with germination related traits. In the present study, a second mapping population developed from a cross between the cultivars ‘Beinhart-1000’ and ‘Hicks’ was investigated.

## Materials and methods

The mapping population was comprised of 118 doubled haploid (DH) lines developed from a cross between the cigar tobacco line ‘Beinhart-1000’ and the flue-cured tobacco cultivar ‘Hicks’ (Vontimitta and Lewis 2012). Seeds of the DH lines were produced in a greenhouse in Raleigh, NC, USA, during the year 2014. After harvest seed material was stored under ambient laboratory temperatures in seed packets, but under conditions of low relative humidity (~ 30%). Germination experiments were performed in 2016. The lines were genotyped with 256 microsatellite markers distributed among 24 linkage groups. Four germination-related traits were investigated by examining seeds either untreated (C) or after a moderate controlled deterioration (CD): total germination (TG; visible radical emergence in %), normal germination (NG; appearance of normal seedlings (ISTA 2017) in %), time to reach 50% of total germination (TG; h), and the area under the curve (AUC; the integration of the fitted curve between *t* = 0 and a defined endpoint *t* = *x*) after 200 h of germination.

Seed germination for C and after CD was performed for each entry in four replications of 50 seeds per line on moistened filter paper (90 mm round filter, C 160; Munktell & FILTRAK GmbH, Germany) in Petri dishes placed in a climate chamber (Panasonic MLR-352-PE). The temperatures were 30 ± 2 °C and 20 ± 2 °C, during illumination (8 h) and darkness (16 h), respectively.

In order to determine the germination speed, germinated seeds (total germination) were counted daily within a germination period of 10 days. Based on these counts, the time needed to reach 50% of TG and the AUC were determined using germination software GERMINATOR (Joosen et al. 2010). On day 10, TG and NG were recorded.

For CD, seeds were placed into permeable paper bags and rehydrated in a climate chamber (22 ± 2 °C; 10% relative humidity) to have identical initial moisture content before treatment. Afterwards, the bags with seeds were placed on plastic racks, which were positioned in airtight boxes containing 1 L of unsaturated LiCl solution (30 g LiCl/100 ml H_2_O), to reach RH of 60% inside the boxes. The boxes were placed in aging chambers at a temperature of 45 °C for 30 days.

For QTL analysis, we used mean values of the traits under investigation applying the method inclusive composite interval mapping (ICIM) command implemented by *IciMapping 4.2.53* (http://www.isbreeding.net/ (released in September 2019)), in order to detect putative additive QTLs, where the walking speed chosen for all QTL was 1.0 cM. A LOD score of > 2.0 ≤ 3 was applied to detect QTLs as significant and > 3.0 as highly significant. ICIM was chosen over composite interval mapping (CIM) implemented in other QTL mapping softwares like *QTLCartographer* as it avoids the possible increase of sampling variance and the complicated background marker selection process implemented in CIM (Meng et al. 2015).

## Results and discussion

The germplasm showed continuous variation for all the traits investigated (Table S1, Fig. S1). The parents ‘Beinhart-1000’ and ‘Hicks’ differed slightly in TG-C reaching to 87.9 and 93.5% whereas CD decreased their values to 83.5 and 84.2%, respectively. For the speed of germination, the differences were even more pronounced. ‘Beinhart-1000’ and ‘Hicks reached’ for the trait AUC values of 88.3 and 108.3 under control as well as 80.2 and 89.4 after CD, respectively. Skewness of TG and NS towards the right side is evident indicating that the initial performance of seed germination was very high (Table S1) whereas CD also did not impose any effect the mean performance as far as TG and NG were concerned. On the other hand, T50 and AUC followed a normal distribution where T50 was increased considerably under CD. On the contrary, AUC was reduced for CD. This phenotypic distribution enabled us to perform the QTL analysis for potential loci underlying these traits.

**Fig. 1:**
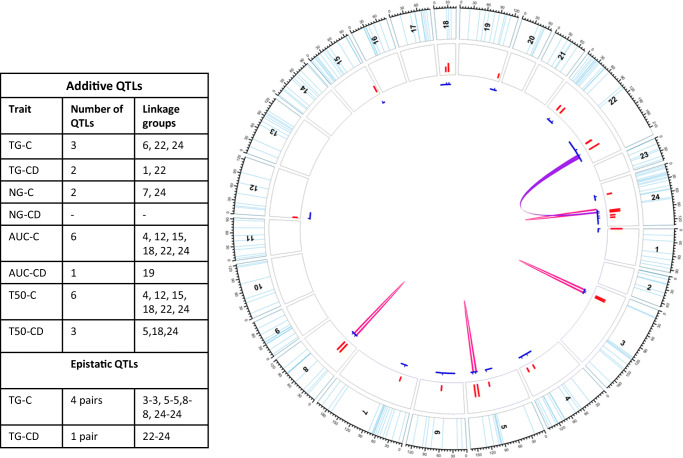
Distribution of additive (unconnected blue lines in the inner circle) and epistatic (connected blue lines in inner circle) QTLs. Skyblue lines in the outer track indicate the marker positions on each chromosome; red bars in the second circle indicate the LOD values of QTLs. The blue lines under the track circle indicate the confidence interval of QTLs with small vertical lines point to the peak position. The colored lines linked different biallelic epistasic QTLs (pink = TG under control; purple = TG after controlled deterioration. Table on the left shows number of additive and epistatic QTLs detected (for reference Tables S2 (Additive QTLs) and S3 (Epistatic QTLs

Additive QTLs were found for all four traits considered on eleven different linkage groups (LGs): 1, 4, 5, 6, 7, 12, 15, 18, 19, 22, and 24 (Fig. 1, Table S2). Individually, LG 24 carried the most loci (5 QTLs) for the investigated traits, albeit at different intervals. A total of four QTLs were discovered on LG 22 and LG 18 carrying 3 QTLs. Each of LG 4, 12, and 15 carried two QTLs. In the end, one QTL was discovered on each of LGs 1, 5, 6, 7, and 19. From individual traits perspective, each of AUC-C and T50-C was controlled by six QTLs distributed on LGs 4, 12, 15, 18, 22, and 24 (LOD = 2.02–4.18 and phenotypic variation explained (PVE) = 3.29–18.76). All the six QTLs of both AUC-C and T50-C overlapped with each other with similar LOD and PVE values. But more contrastingly, they were contributed by opposite parents.

Three QTLs were discovered for TG-C on LG 6, 22 and 24 (LOD = 2.01–3.4, PVE = 7.17–13.53) whereas two QTLs were discovered for NS-C on LGs 7 and 24 (LOD = 2.2–2.29, PVE = 8.63–9.41) where LG24 carried overlapped QTLs for TG-C and NS-C. Two QTLs were found on LGs 1 and 22 for TG-CD (LOD = 2.89–3.08, PVE = 11.52–12.32). Furthermore, another three LGs carried three QTLs linked with T50-CD (LOD = 2.05–2.33, PVE = 3.8–7.97) whereas only one QTL was discovered for AUC-CD (LOD = 2.03, PVE = 7.5) on LG 19. No QTL, however, could be detected for NG-CD.

With regard to epistatic QTLs, a total of four and one pair was discovered for TG-C and TG-S, respectively (Fig. 1, Table S3) whereas no epistatic QTLs could be detected for other traits. The four pairs linked with TG-C involved nearby loci located on similar LGs viz. LGs 3-3, 5-5, 8-8 and 24-24 (LOD = 5.12–6.20) that explained an addition 6.47–7.55% variation. The only pair linked with TG-CD involved LGs 22-24 (LOD = 5.70) responsible for another 22.13% variation.

In tobacco, there is only one previously published genetic study on seed germination (Agacka-Mołdoch et al. 2015). It was based on a cross between the cultivars ‘Florida 301’ and ‘Hicks.’ Comparable to the present investigations, ‘Hicks’ exhibited higher germination (TG) and germination speed (AUC) under control compared to the parent ‘Florida 301.’ However, this was equalised after the CD treatment. In both studies, both parents contributed favourable alleles for the traits under investigation at different loci.

Overall, the mapping results of the present study confirm former studies in several plant species that traits related to germination are very complex and under polygenic control. In durum wheat (*Triticum durum* L.), Rehman Arif and Börner (2019) investigated a bi-parental mapping population. QTLs for initial germination, germination after accelerated aging treatment and relative germination were distributed on 6 out of 14 chromosomes. In an oilseed rape (*Brassica napus* L.; 19 chromosomes), bi-parental mapping population QTL for seed longevity were detected on 7 linkage groups (Nagel et al. 2011). After performing genome wide association studies, even more loci became detectable as demonstrated for hexaploid wheat (*Triticum aestivum* L.) by Rehman Arif and Börner (2020). Loci for seed longevity were identified on 14 of the 21 chromosomes verifying the high complexity of germination traits.

In tobacco, more investigations are needed exploiting bi-parental and association mapping populations with high marker coverage in order to further elucidate germination-related traits and to identify candidate genes. This will be of special interest for seed industry but also genebank curators.

## Supplementary Information

ESM 1 (PPTX 3653 kb)

ESM 2 (XLSX 12 kb)

ESM 3 (XLSX 13 kb)

ESM 4 (XLSX 12 kb)
