# Supplementary material for: QTL analysis of seed germination traits in tobacco (Nicotiana tabacum L.)
Source: J Appl Genet. 2021 Mar 5;62(3):441–4. doi: 10.1007/s13353-021-00623-6 (PMC8357679; doi:10.1007/s13353-021-00623-6)
Supplement: Supplementary file 1 — (PPTX 3653 kb) [file 13353_2021_623_MOESM1_ESM.pptx]

## Slide 1
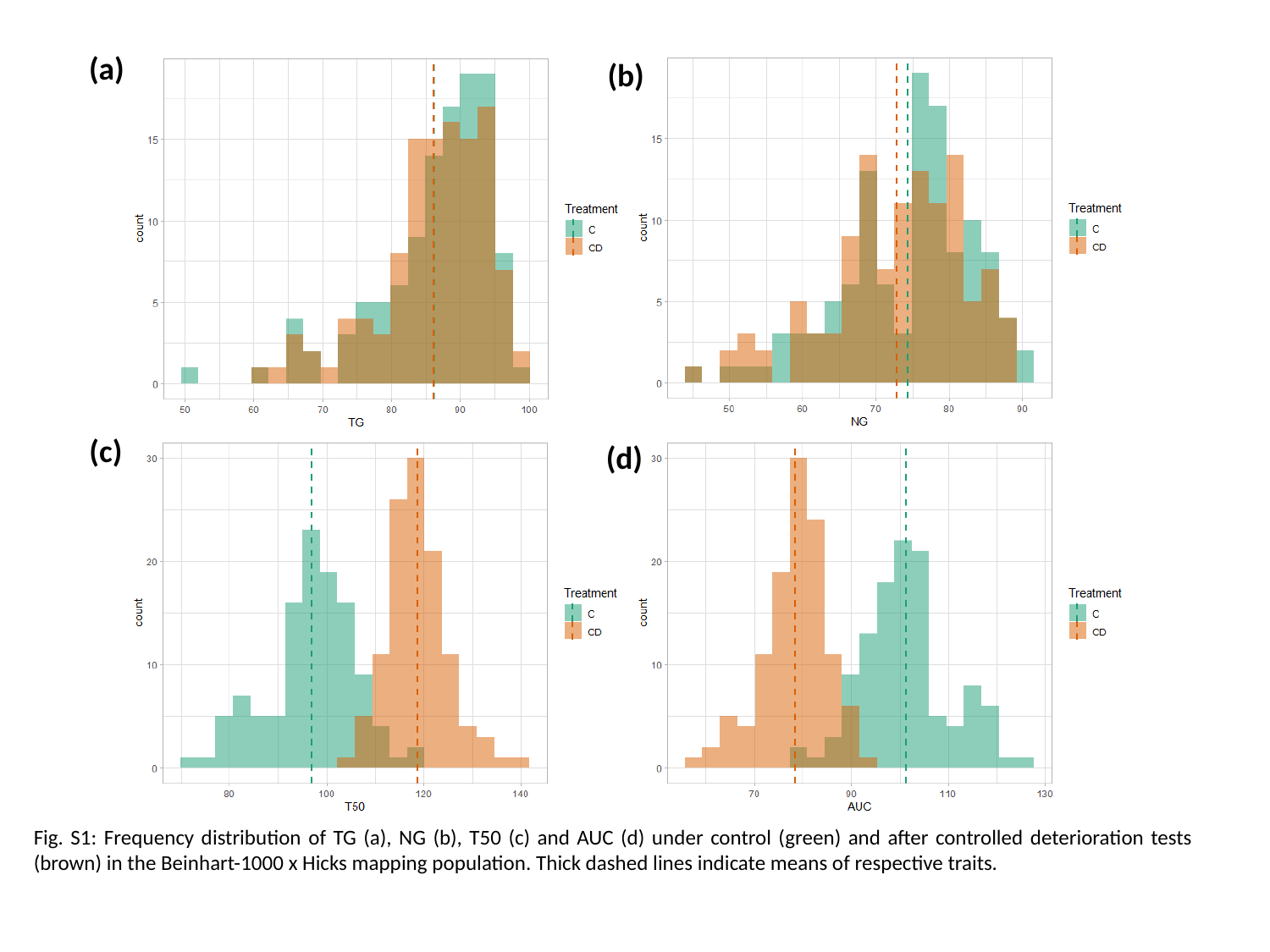

(a)
(b)
(c)
(d)
Fig. S1: Frequency distribution of TG (a), NG (b), T50 (c) and AUC (d) under control (green) and after controlled deterioration tests (brown) in the Beinhart-1000 x Hicks mapping population. Thick dashed lines indicate means of respective traits.
